# „Die passende Chemie wäre entscheidend … wenn man denn eine Wahl hätte“ – eine qualitative Studie zu Präferenzen von Therapiesuchenden einer ambulanten Psychotherapie

**DOI:** 10.1007/s00278-023-00656-8

**Published:** 2023-04-27

**Authors:** Vanessa Pantle, Lena Maier, Ileana Schmalbach, Deborah Engesser, Klaus Lieb, Hauke Felix Wiegand, Susanne Singer

**Affiliations:** 1 Klinik für Psychiatrie und Psychotherapie, Universitätsmedizin der Johannes Gutenberg-Universität Mainz, Untere Zahlbacher Str. 8, 55131 Mainz, Deutschland; 2 Abteilung Epidemiologie und Versorgungsforschung, Institut für Medizinische Biometrie, Epidemiologie und Informatik (IMBEI), Universitätsmedizin Mainz, Mainz, Deutschland; 3 Abteilung Medizinische Psychologie und Medizinische Soziologie, Universitätsmedizin Mainz, Mainz, Deutschland

**Keywords:** Versorgungsforschung, Patient:innenperspektive, Suchprozess, Hürden, Qualitative Interviews, Health Services Research, Clients’ perspective, Search process, Barriers, Qualitative interviews

## Abstract

**Hintergrund und Fragestellung:**

Mit der Psychotherapiestrukturreform von 2017 sollte u.a. der Zugang zu ambulanter Psychotherapie erleichtert werden. Ein Aspekt der Evaluation der Reform ist, die Perspektive der Therapiesuchenden abzubilden. Der Fokus der vorliegenden Studie liegt dabei auf der Frage, welche Präferenzen Therapiesuchende äußern und inwiefern sie diese in den Suchprozess einbringen können.

**Methoden:**

Mit Hilfe von qualitativen, leitfadengestützten Interviews wurden Therapiesuchende zu Beginn ihrer Suche (erster Erhebungszeitpunkt = t1) zu ihren Präferenzen und drei Monate später (zweiter Erhebungszeitpunkt = t2) nach ihren Erfahrungen befragt. Im Rahmen einer strukturierten Inhaltsanalyse wurden die Transkripte zunächst kategorisiert, bevor Generalisierungen, welche die Kernaussagen der Interviews abbilden, abgeleitet wurden.

**Ergebnisse:**

Es wurden 83 Interviews mit 46 Therapiesuchenden geführt. Mehr als die Hälfte gaben an, dass sie anfängliche Präferenzen im Verlauf der Suche nicht länger priorisierten, um so die Chance auf einen Therapieplatz zu erhöhen. Vor der Suche lag der Fokus darauf, dass „die Chemie“ zu dem Therapeuten/der Therapeutin stimmt; nach bzw. während der Suche war z. B. die Vereinbarkeit mit dem Alltag entscheidender.

**Diskussion:**

Die Studie zeigt, dass es weiter nötig ist, den Zugang zu Psychotherapie zu vereinfachen und die grundlegende Versorgungssituation zu verbessern. Um gute Voraussetzungen für den Therapieprozess und die Behandlungsergebnisse zu schaffen, wäre es erforderlich, den Therapiesuchenden die Berücksichtigung ihrer Präferenzen zu ermöglichen.

## Abstract (en)

**Background and research question:**

One of the aims of the German psychotherapy structural reform in 2017 was to facilitate access to outpatient psychotherapy. One aspect of the evaluation of the reform was to illustrate the clients’ perspective regarding the search process. The study at hand focuses on the extent to which clients are able to include their preferences in the search process.

**Methods:**

Clients were interviewed about their preferences prior to searching for outpatient psychotherapy (first date of survey = t1) and about their experiences 3 months later (second date of survey = t2). For this purpose, qualitative, guideline-based interviews were used. Within the framework of a structured content analysis, the transcripts were first categorized before generalizations were derived that depict the core statements of the interviews.

**Results:**

A total of 83 interviews with 46 clients were conducted. More than half of them stated that they no longer prioritized initial preferences during the search in order to increase their chance of psychotherapy treatment. Before the search, the focus was on the “chemistry” between themselves and the therapist and after or during the search compatibility with everyday life, for example, was more decisive.

**Discussion:**

The study demonstrates that there is a further need to simplify access to psychotherapy and to improve the basic care situation. To create good conditions for the therapy process and treatment outcomes, it would be necessary to enable clients to give consideration to their preferences.

## Hinführung zum Thema

Eine ambulante Psychotherapie ist aktuell gefragter denn je (Deutsche Psychotherapeuten Vereinigung 2021). Die Suche gestaltet sich mühsam; die Wartezeiten sind lang, freie Plätze selten (Singer et al. 2021). Obgleich die Behandlungsleitlinien für viele psychische Erkrankungen eine ambulante Psychotherapie empfehlen, kann diese in einem Großteil der Fälle nicht zeitnah angeboten werden (z. B. Wiegand et al. 2020). Dieser Missstand führt zu einer Art Konkurrenz um die vorhandenen Plätze.

Ein Teil der Evaluation der Psychotherapiestrukturreform sollte die Perspektiven von Therapiesuchenden abbilden. Fokus der vorliegenden Studie war es, Therapiepräferenzen zu identifizieren und zu erfassen, inwiefern diese über den Suchprozess hinweg berücksichtigt werden konnten. Dazu befragten wir Therapiesuchende mit qualitativen Interviews vor dem Beginn ihrer Suche und erneut 3 Monate später.

## Einleitung

Unter „Therapiepräferenzen“ können im weiteren Sinne „Verhaltensweisen oder Eigenschaften, die Therapiesuchende schätzen oder sich von Therapeut:innen wünschen würden,“ verstanden werden (Glass et al. 2001). Davon abzugrenzen sind Erwartungen, die Therapiesuchende an die Behandlung oder die Therapeut:innen haben können, welche eher spiegeln, was in der Therapie tatsächlich passieren wird oder passieren sollte (Swift et al. 2011).

Im Hinblick auf die Präferenzen von Therapiesuchenden kann angenommen werden, dass sich deren Berücksichtigung z. B. positiv auf die therapeutische Allianz, die Initialisierung der Therapie (Gelhorn et al. 2011) oder auch das Therapie-Commitment (Swift et al. 2021) auswirken kann. Auch zeigen Metaanalysen und Studien einen Zusammenhang mit besseren Therapieergebnissen und -erfolgen (Gelhorn et al. 2011; Swift et al. 2021; Lindhiem et al. 2014; Williams et al. 2016). Werden konkrete Therapiepräferenzen hingegen nicht berücksichtigt, profitieren Patient:innen im subjektiven Erleben weniger von der Therapie (Williams et al. 2016). Zusätzlich vermuten Swift und Kolleg:innen, dass auch die Wahlmöglichkeit hinsichtlich der Behandlung einen positiven Einfluss auf die Therapiemotivation haben könnte (Swift et al. 2021). Bestenfalls könnten sich Therapiesuchende also im Rahmen ihrer Präferenzen ihre/n Therapeutin/en aussuchen.

Die Berücksichtigung der Präferenzen der Therapiesuchenden kann folglich als relevant für den therapeutischen Prozess angenommen werden und sollte als Einflussfaktor auf die Wirksamkeit der Therapie bereits im Rahmen des Suchprozesses und im Sinne einer leitliniengerechten Behandlung psychischer Erkrankungen ermöglicht werden (z. B. Deutsche Gesellschaft für Psychiatrie und Psychotherapie, Psychosomatik und Nervenheilkunde et al. 2015).

Hinsichtlich der Art der Präferenzen unterscheiden z. B. Swift und Kolleg:innen Aktivitätspräferenzen (z. B. inwiefern Therapeut:innen eine eher aktive oder eine eher zuhörende Rolle einnehmen sollen), Therapeutinnen:präferenzen (z. B. hinsichtlich Erfahrung oder Ethnizität) und Behandlungspräferenzen (bezüglich der verschiedenen Therapieformen) (Swift et al. 2021). Unabhängig von dieser Differenzierung berichten andere Studien, dass z. B. die Nähe zum Wohnort, ein bestimmtes Therapieverfahren oder das Geschlecht der Therapeutin/des Therapeuten als Präferenzen genannt werden (Felber et al. 2005). Albani et al. (2010) eruierten hingegen, dass 73 % der Befragten keine Präferenz bzgl. des Therapeut:innen-Geschlechts und 85 % keine Präferenz hinsichtlich des Alters der Therapeutin/des Therapeuten äußerten. Ergänzend dazu seien während des ersten persönlichen Kontakts „Sympathie, Empathie und der Eindruck von Kompetenz“ entscheidend dafür gewesen, eine Psychotherapie zu beginnen (Felber et al. 2005).

Obgleich Studien die Relevanz des Einbezugs der Präferenzen belegen, ist unklar, inwiefern sich dies in der Routineversorgung in Deutschland umsetzen lässt. Ziel der Studie war daher, Therapiesuchende zeitgleich zu ihrer Suche (vor dem Beginn und 3 Monate später) zu Präferenzen zu befragen und zu erfassen, inwiefern diese während des Suchprozesses berücksichtigt werden können.

## Methode

### Studiendesign, Kontaktaufnahme und Erhebung

Es handelt sich um eine qualitative prospektive Studie, welche im Rahmen der vom Gemeinsamen Bundesausschuss beauftragten Evaluation der Psychotherapiestrukturreform von 2017 durchgeführt wurde. Dabei bildet die Untersuchung der aktuellen Zugangs- und Versorgungsprobleme aus der Perspektive der Therapiesuchenden in dem vorliegenden Artikel einen Teilaspekt dieser Evaluation ab. Die Befragung der Therapiesuchenden erfolgte mittels qualitativen Interviews zu 2 Untersuchungszeitpunkten im Abstand von 3 Monaten. Um möglichst unvoreingenommene Vorstellungen der Therapiesuchenden zu erfassen, wurden vorerst nur Therapiesuchende interviewt, die entweder noch nie eine Psychotherapie in Anspruch genommen hatten oder deren vergangene Therapie vor der Reform 2017 abgeschlossen war. Der Großteil der Therapiesuchenden wurde dabei sowohl zu ihren Erwartungen bezüglich der Suche (t1) als auch zu ihren Erfahrungen mit der Suche 3 Monate später (t2) befragt. Aufgrund der COVID-19-bedingten Restriktionen (z. B. Schließung der Praxen) wurde die Anwerbung jedoch so erschwert, dass das ursprüngliche Ausschlusskriterium (therapeutische Vorerfahrung) gelockert wurde: Ergänzend konnten 8 weitere Therapiesuchende in die Studie inkludiert werden, welche zum Zeitpunkt der Studie bereits in ambulanter psychotherapeutischer Behandlung waren. Diese wurden daher nur einmalig (t2) retrospektiv befragt. Ausschlusskriterien waren akute psychotische Episoden, demenzielle Erkrankungen und ein Alter < 18 Jahre. Die Ansprache der Therapiesuchenden erfolgte sowohl über die Ärzt:innen der Klinik für Psychiatrie und Psychotherapie als auch über die Notaufnahme der Universitätsmedizin Mainz sowie über Zuweiser:innen, z. B. Hausärzt:innen. Die Teilnahme an der Studie wurde für je 30 min Zeitaufwand mit 25 € vergütet. Alle Therapiesuchende wurden zu Beginn ausführlich über Studieninhalte und Datenschutz aufgeklärt und nach schriftlicher Einwilligung in die Studie eingeschlossen.

Der Erhebungszeitraum war nach etwa einem Jahr abgeschlossen, wobei das erste Interview am 31.07.2020 und das letzte am 13.08.2021 geführt wurde. Ein Positivvotum der Landesärztekammer Rheinland-Pfalz liegt vor.

### Zusammensetzung der Studiengruppe

Die Interviews wurden von 3 psychologischen Mitarbeiterinnen (VP, LM, IS) und einer Mitarbeiterin mit epidemiologischer Ausbildung (DE) persönlich oder telefonisch durchgeführt. Zwei der psychologischen Mitarbeiterinnen befanden sich während der Studiendurchführung in der Ausbildung zur Psychotherapeutin (VP, LM). Die Studienleitung (SS) ist approbierte Psychotherapeutin. Weitere Mitglieder der Studiengruppe (KL, HFW) sind Fachärzte für Psychiatrie und Psychotherapie. 6 der 7 Autor:innen haben durch ihre berufliche Tätigkeit Kontakt zu Therapiesuchenden: KL, HFW und VP stehen dabei im Austausch mit Patient:innen der Klinik für Psychiatrie und Psychotherapie, die auf der Suche nach einer ambulanten Psychotherapie sind und sowohl von ihren Erwartungen an die Suche als auch von bereits gemachten Erfahrungen berichten.

Alle Autor:innen haben – auch durch die professionsspezifische Selbsterfahrung der Aus- bzw. Weiterbildung – im weiteren Sinne Erfahrung mit Psychotherapie. Aufgrund ihrer klinischen Tätigkeit haben die Autor:innen häufig erfahren, dass es für Ratsuchende, v. a. angesichts des Rechtes auf „freie Arztwahl“, schwierig (bis unmöglich) ist, adäquate Behandlungsangebote zu erhalten. Das bedeutet, dass die Autor:innen – ebenso wie der Auftraggeber der Studie, der Innovationsfonds des Gemeinsamen Bundesausschusses – bei der Planung und Durchführung der Studie gewisse Interessen verfolgten, nämlich den Zugang zur ambulanten Psychotherapie zu verbessern.

Um das Risiko möglicher Verzerrungen der Studienergebnisse durch diese Interessen zu verringern, wurden die Interviewerinnen vor Beginn der Datenerhebung in der Interviewdurchführung geschult und standen im weiteren Forschungsprozess in engem intervisorischen Austausch. Die Leitfragen für das Interview wurden dabei möglichst offen und wertungsfrei formuliert. Darüber hinaus wurde bei der Zusammensetzung der Studiengruppe darauf geachtet, dass verschiedene professionelle Hintergründe vertreten waren.

### Leitfaden

Als theoretischer Hintergrund für die Erstellung des Leitfadens wurden bisherige Forschungsbefunde zur aktuellen Versorgungs- und Zugangsproblematik hinsichtlich der ambulanten Psychotherapieversorgung in Deutschland herangezogen (Singer et al. 2021; Wiegand et al. 2020). Der erste Teil des Interviews begann narrativ mit einer Erzählaufforderung, um die explorativen Eigenschaften der qualitativen Analyse zu fördern (z. B. t1 *„Man hat Ihnen empfohlen, eine ambulante Psychotherapie zu beginnen; Wenn Sie an die Suche denken, was geht Ihnen durch den Kopf?“*). Für den darauffolgenden Leitfaden wurden vorab Fragen und relevante Themenkomplexe diskutiert und zusammengeführt. Darüber konnten grundlegende Aspekte des Suchprozesses (z. B. „geplantes Vorgehen bei der Suche“) erfasst werden. Der vorliegende Artikel fokussiert dabei thematische Bezüge hinsichtlich der Therapiepräferenzen (zu t1 *„Was wäre Ihnen bei Ihrem/r Therapeuten/in wichtig?“* und *„Inwiefern spielen diese Präferenzen eine Rolle bei der Suche?“*; zu t2 *„Wir haben im ersten Interview darüber gesprochen, was Ihnen bei der Therapeutensuche wichtig ist; inwiefern konnten Sie diese Präferenzen berücksichtigen?“*). Für die 8 Therapiesuchenden, welche nur einmalig befragt wurden, wurde eine angepasste Version des Leifadens genutzt, in welchem die Leitfragen aus der ersten Befragung nun so gestellt wurden, dass sie die gemachten Erfahrungen erfassen konnten (*„Wenn Sie an die Suche zurückdenken: Was war Ihnen bei Ihrem/Ihrer Therapeuten/in wichtig? Inwiefern konnten Sie die Präferenzen bei der Suche berücksichtigen?“*). Des Weiteren wurden über einen Fragebogen soziodemografische Daten wie Alter und Geschlecht sowie aktuelle Beschwerden erhoben. Zusätzliche Anmerkungen, Auffälligkeiten oder Verhaltensbeobachtungen wurden in einem Beobachtungsprotokoll dokumentiert.

### Auswertung

Die Interviews wurden nach vorheriger Zustimmung per Diktiergerät aufgezeichnet und anschließend durch einen externen Dienstleister transkribiert. Diese Transkripte wurden in die Software MAXQDA (2020) zur qualitativen Datenanalyse eingepflegt. Die Auswertung der Daten wurde dabei in Anlehnung an die qualitative Inhaltsanalyse nach Mayring (Mayring und Fenzl 2019) durchgeführt. Dabei entwickelten zwei geschulte Mitarbeiterinnen unabhängig voneinander einen Codebaum (Kategoriensystem) und einen Codierleitfaden. Ein Drittel der Interviews wurde zunächst von zwei Mitarbeiterinnen unabhängig voneinander doppelt gesichtet und analysiert. In Anlehnung an den Leitfaden wurden daraus Kategorien gebildet. Um ein hohes Maß an Übereinstimmung zu erreichen, wurden Unterschiede in der Kategorienbildung im Forscherteam kontinuierlich diskutiert, bis ein Konsens gefunden wurde. Dabei wurde sowohl deduktiv anhand der Leitfragen (*„Was wäre Ihnen bei Ihrem/r Therapeuten/in wichtig?“*) als auch induktiv anhand des Materials kategorisiert, wobei Mehrfachzuordnungen möglich waren. Abweichungen wurden anschließend erneut konsensuell überprüft und modifiziert. Auf Basis dessen wurden ein finaler Codebaum erarbeitet und ein Codierleitfaden entworfen, welcher in seiner endgültigen Version für die Auswertung aller Interviews Anwendung fand. Die Inhalte dieser Unterkategorien wurden jeweils ausgewertet und zusammengefasst, um daraus übergreifende Generalisierungen bilden zu können (Dittrich et al. 2021).

## Ergebnisse

### Stichprobencharakteristika

Insgesamt nahmen 49 Therapiesuchende an der Studie teil; 3 Therapiesuchende wurden jedoch im Nachhinein von der Studie ausgeschlossen: Ein Patient, weil er zum Zeitpunkt des Interviews akut psychotisch exazerbiert war, und 2 Therapiesuchende, weil sie ihr Einverständnis zur Studie ohne Angabe weiterer Gründe widerriefen. Daraus ergibt sich eine Gesamtteilnehmerzahl von 46 Probanden, von denen 37 jeweils zu t1 und t2 interviewt wurden, wobei ein/e Therapiesuchende/r zu t2 „lost to follow-up“ war, sodass nur das Interview zu t1 ausgewertet wurde. Ergänzend dazu berichteten 8 Therapiesuchende einmalig retrospektiv (nur zu t2). Insgesamt wurden 83 Interviews ausgewertet; eine theoretische Sättigung der Antworten wurde erreicht.

Dabei wurden 41 % der Therapiesuchenden während und nach ihrer Behandlung in der Klinik für Psychiatrie und Psychotherapie interviewt (alle jeweils zu t1 und t2), weitere 41 % kontaktieren das Studienteam über ihre Zuweiser (7 davon berichteten retrospektiv), und 18 % der Therapiesuchenden wurden nach einem Kontakt zur medizinischen Notaufnahme befragt (wovon ein Patient ebenfalls nur retrospektiv berichtete).

Das durchschnittliche Alter betrug 40 Jahre (*SD* ± 15); 54 % der Therapiesuchenden waren weiblichen, 46 % männlichen Geschlechts. „Depressive Beschwerden“ wurden von 80 % der Therapiesuchenden berichtet. Ängste und Panikattacken beklagten 57 % der Therapiesuchenden. Schwierigkeiten in sozialen Interaktionen wurden von 24 % als problematisch thematisiert. Somatische Beschwerden wurden von 22 % der Therapiesuchenden angegeben. Stress, Suizidgedanken, erlebte Traumata, Ess- und Zwangsstörungen, Konzentrationsschwierigkeiten und Substanzabhängigkeiten wurden als weitere Belastungen genannt, wobei Mehrfachnennungen grundsätzlich möglich waren.

### Interviewmaterial

Die Interviews hatten, zusammen genommen, eine Dauer von 23,5 h, wobei das erste Interview im Durchschnitt 17 min, das zweite Interview im Durchschnitt 19 min dauerte. Das kürzeste Interview dauerte 7 min, das längste 45 min.

### Präferenzen vor Beginn der Suche

Während des ersten Interviews konnten die Aussagen der Therapiesuchenden 11 Unterkategorien (*„passende Chemie/Sympathie“, „therapeutische Kompetenzen“, „Interventionen/Problembewältigung“, Geschlechtspräferenz, Therapieverfahren, „Alltagskompatibilität“, Alterspräferenz, Kassensitz/Approbation, keine Präferenz, Praxisräume, Sonstiges*) zugeordnet werden. In Tab. 1 werden dabei sowohl die Anzahl der Therapiesuchenden, welche die Präferenz äußerten, als auch die Häufigkeit der Äußerungen zu der Präferenz abgebildet.

**Table 1:** 

Kategorie	Anzahl Therapiesuchende	Anzahl Nennungen
Therapeutische Kompetenzen	15	18
Interventionen/Problembewältigung	15	18
Geschlecht	12	15
Therapieverfahren	11	13
Alltagskompatibilität	6	6
Alter	5	6
Kassensitz/Approbation	4	5
Keine Präferenz	4	4
Praxisräume	2	3
Sonstiges	2	2

Es wurde von 19 Therapiesuchenden betont, dass die **„passende Chemie“** zwischen ihnen und dem/der Therapeut:in für sie relevant sei (*„Mir ist es wichtig, dass die Chemie stimmt, aber das weiß man vorher nicht, man muss einfach zum Termin hin und ggf. dann den Therapeuten wechseln“* – *ID218*). Bestimmte **„therapeutische Kompetenzen“** (z. B. Vertrauen, Sympathie) wünschten sich 15 Therapiesuchende *(„Ich wünsche mir jmd., dem ich persönliche Dinge anvertrauen kann. Dinge, die ich nicht unbedingt über mich preisgeben möchte. Es müsste jmd. sein, dem ich vertrauen kann, der gut zuhören kann und der auch sympathisch ist.“ – ID211).* Darüber hinaus hofften 15 Therapiesuchende auf **„konkrete Problemlösestrategien“** *(„Also ich erwarte, dass mein Therapeut gut analysiert, wo das Problem liegt. Und mir hilft, eine Struktur zu finden. Also, warum ich in diese Erkrankung gefallen bin. Dass er mir Lösungen anbietet, nicht nur Kritik oder Analyse, sondern auch Lösungen, wie ich da rauskommen kann“ – ID116)*. Hinsichtlich der **Geschlechtspräferenz** äußerten sich 12 Therapiesuchende, wobei 4 Patienten und eine Patientin angaben, dass ihnen das Geschlecht egal sei *(„Ich habe, was das Geschlecht angeht, keine Präferenzen. Das ist relativ egal.“ – ID120). *Jeweils 3 Patientinnen und 3 Patienten würden eine Therapeutin präferieren. Ein Patient suchte explizit einen männlichen Therapeuten.

Bezüglich des **Therapieverfahrens** gaben 7 Therapiesuchende an, dass sie auf der Suche nach einer Verhaltenstherapie seien *(„Auf jeden Fall Verhaltenstherapie“ – ID313)*, 3 Therapiesuchende präferierten eine tiefenpsychologische Therapie *(„Also ich will einen Therapeuten haben, die tiefenpsychologisch mit mir was macht.“ – ID314)*, und eine Aussage galt nur der priorisierten Suche nach einem spezifischen Therapieverfahren, ohne zu äußern, um welches Verfahren es sich handeln solle.

Weiterhin relevant schien die **„Alltagskompatibilität“**, also die Vereinbarkeit von Beruf und Alltag mit der Therapie, was sich z. B. über einen individuellen Entfernungsradius darstellt, in welchem 6 Therapiesuchende bevorzugt nach einer Therapie suchen wollten (*„Und es wäre mir halt noch wichtig, dass die Termine auch so gesetzt sind, dass ich noch ein normales Arbeitsleben führen kann“ – ID320*).

**Alterspräferenzen** wurden von 5 Therapiesuchenden berichtet, wobei 2 Therapiesuchende ältere *(„Ich denke mal, ein junger Therapeut wäre jetzt auch nicht so geeignet wie jemand, der vielleicht etwas älter ist“ – ID213)*, 2 Therapiesuchende jüngere Therapeut:innen bevorzugten *(„Ehrlich gesagt, dass er nicht so alt ist“ – ID306)*. Ein Therapiesuchender merkte an, dass das Alter für ihn keine Rolle spielen würde.

Die Abrechnung über einen **Kassensitz und die „fachkundig spezifische Approbation“** der Therapeut:innen seien für 4 Therapiesuchende relevant *(„Naja, er sollte eine Zulassung haben. Also, Arzt oder Psychologe sein.“ – ID113).*

Weitere 4 Therapiesuchende äußerten, **keine Präferenzen** zu haben *(„Nein [ich habe keine Präferenzen]. Hauptsache, ich bekomme einen Therapeuten.“ – ID313).*

Von klaren Vorstellungen bezüglich der **Praxisräume** berichteten 2 Therapiesuchende *(„Und die Praxis. Auch die sollten ‚klar‘ gestaltet sein. Kein ‚Schnickschnack‘ oder Deko oder so. Es soll Geborgenheit vermitteln, aber auch keine Ablenkung im Raum bestehen. Weniger kann manchmal mehr sein. Ich will da keine Urkunden oder so sehen.“ – ID113)*.

**Sonstige Präferenzen**, wie z. B. Englisch als Therapiesprache oder die Beurteilung der Therapeut:innen anhand ihrer Namen, wurden jeweils von einer Person genannt *(„Ist vielleicht ein bisschen unlogisch, aber ich gehe oft nach den Namen. Ob der Name mir gefällt.“ – ID110).*

Zu ergänzen ist, dass 8 Therapiesuchende nur über **unzureichende Grundkenntnisse** berichteten (z. B. im Hinblick auf die verschiedenen Therapieverfahren), wodurch die Suche gar nicht habe spezifiziert werden können *(„Aber mit diesen spezifischen Psychotherapieformen kenne ich mich jetzt auch nicht aus. Und das war ebenso das Nächste, wo ich dachte, keine Ahnung, was ich davon jetzt als Suchanfrage überhaupt angeben soll“ – ID318).*

### Präferenzen 3 Monate später

Im zweiten Interview erklärten 14 Therapiesuchende (31 %), dass sie eine Psychotherapie beginnen konnten oder einen Platz in Aussicht hatten. Im Hinblick auf die Therapiepräferenzen gaben 15 Therapiesuchende an, ihre Präferenzen im Suchprozess berücksichtigt zu haben (Tab. 2). Dabei stand bei 9 Therapiesuchenden die **Alltagskompatibilität **im Fokus* („Die räumlichen Nähe, dass ich jetzt nicht eine Stunde fahren muss, aber so im Großraum … habe ich mich im Prinzip auf alle möglichen Therapeuten gemeldet.“ – ID120)*. Ein gutes Bauchgefühl bzgl. **passender Chemie **sei für 5 Therapiesuchende bei der Suche relevant gewesen, wobei dies z. B. am Klang der Stimme während der Terminvereinbarung beurteilt wurde *(„Ich habe mit der Therapeutin selbst gesprochen. Diese Stimme und so, das passte sofort. Also das war nichts Unangenehmes. Ich habe da schon sehr auf meinen Bauch gehört.“ – ID103).*

**Table 2:** 

Kategorie	Anzahl Therapiesuchende	Anzahl Nennungen
Alltagskompatibilität	9	9
Passende Chemie/Sympathie	5	5
Therapieverfahren	4	5
Geschlecht	3	3
Bewertung	1	1
Alter	1	1

Ein bestimmtes **Therapieverfahren** war für 4 Therapiesuchende von Relevanz *(„Ich habe meine Verhaltenstherapie bekommen. Da kann ich eigentlich nicht meckern.“ – ID104)*. Für 3 Therapiesuchende sei das **Geschlecht** wichtig gewesen *(„Und es ist auch eine Frau. Was mir halt auch lieber war.“ – ID117)*, eine Patientin habe sich an **Bewertungen im Internet,** z. B. Google-Rezensionen, orientiert *(„Bei manchen sind die Bewertungen mehr negativ als positiv. Und das hat halt eben auch dazu geführt, dass ich gedacht habe, nein, probierst es erst gar nicht.“ – ID102)*, und für einen Patienten sei es von Bedeutung gewesen, dass der/die Therapeut/in im ähnlichen **Alter** sei *(„Ich hatte ja gesagt, dass es mir wichtig ist, dass die Person vielleicht ein bisschen in meinem Altersbereich ist.“ – ID201).*

18 Therapiesuchende äußerten, die Suche nur zu Beginn nach ihren Präferenzen ausgerichtet zu haben (Tab. 3). Dabei berichteten 10 Therapiesuchende, dass der Wunsch nach „schneller Hilfe/Unterstützung“ priorisiert worden sei und andere Präferenzen deswegen nicht länger als entscheidende Suchkriterien einbezogen wurden (*„Nein, in dem Moment, wo es mir elendig ging, bin ich nicht mehr wählerisch geworden. Ich wollte einfach alles, was mir angeboten wird, annehmen.“ – ID116*)

**Table 3:** 

Kategorie	Anzahl Therapiesuchende	Anzahl Nennungen
Geschlecht	4	5
Therapieverfahren	3	3
Alltagskompatibilität	2	3
Präferenzen verändert	2	2
Passende Chemie/Sympathie	1	1
Alternative Behandlung	1	1

So wurde das **Therapeutengeschlecht** von 4 Therapiesuchenden nicht länger als entscheidungsrelevant betrachtet *(„Ich wollte halt am Anfang eher einen männlichen Psychologen haben. Aber, je mehr ich gemerkt habe, dass ich eigentlich kaum eine Wahl habe, da wurde das immer weniger. Mit der Zeit war mir das Geschlecht egal, Hauptsache, einen Platz bekommen oder Hilfe“ – ID320).* Auch gaben 3 Therapiesuchende an nicht mehr ausschließlich im präferierten **Therapieverfahren** zu suchen *(„[Nach Verhaltenstherapie] war ich erst gegangen. Da war aber das Angebot relativ doch klein. Und dann habe ich gedacht, nein, ich rufe einfach alle an und frage erst hinterher.“ – ID319).* Der **Such-Entfernungsradius (Alltagskompatibilität) **wurde von 2 Therapiesuchenden erweitert *(„Das war mir am Ende so ziemlich egal, wie weit die weg sind, so. Ich wollte nur einen Psychologen.“ – ID313).*

Weitere 2 Therapiesuchende **änderten ihre Präferenzen** während der Suche *(„Ehrlich gesagt, habe ich ganz anders gesucht, als ich gedacht habe. Ich habe mich ein bisschen reingelesen. Also ich habe überlegt, ob diese Traumatherapie, dieses EMDR heißt das, ob das nicht was für mich wäre.“ – ID317).* Eine Patientin äußerte, dass ihr Wunsch nach dem „optimalen Therapeuten“ (**Passende Chemie/Sympathie**) für sie nicht länger realistisch sei *(„Ja, also ich denke nicht mehr, dass ich den Therapeuten finden werde, der einfach optimal für mich wäre.“ – ID311)*, und ein Patient wandte sich einer **alternativen Behandlungsform** zu *(„Ich habe im Internet gesucht, und manche hatten Bilder auf der Webseite. Die Person muss mir sympathisch sein. Aber nach einigen erfolglosen Telefonaten habe ich aufgegeben. Ich habe schneller einen Termin bei meinem Heilpraktiker bekommen.“ – ID211).*

## Diskussion

Die vorliegende Studie untersuchte, inwiefern Therapiesuchende individuelle Präferenzen bezüglich der Therapie oder der Behandler während ihrer Therapieplatz-Suche berücksichtigen können.

Zentrales Ergebnis ist, dass mehr als die Hälfte der Therapiesuchenden ihre ursprünglichen Präferenzen über den Suchverlauf von 3 Monaten hinweg nicht länger als entscheidungsrelevant beurteilten und individuelle Alternativen in Erwägung zogen.

Wesentliches Motiv dafür ist, so die Chance auf einen Psychotherapieplatz zu erhöhen. Dieses Ergebnis ist in dem Kontext zu interpretieren, dass nur knapp ein Drittel der Therapiesuchenden in den Interviews berichtete, nach 3 Monaten Suche eine Psychotherapie begonnen zu haben oder zeitnah beginnen zu können. Obgleich der Sucherfolg nicht das primäre Forschungsziel dieser Studie war, liegt die Interpretation nahe, dass die, auch nach der Psychotherapiestrukturreform 2017, unzureichende Psychotherapie-Versorgungssituation ein wesentlicher Faktor sein dürfte, welcher die ausreichende Berücksichtigung individueller Präferenzen in vielen Fällen verhindert.

Bevor Therapiesuchende sich auf die Suche nach einer ambulanten Psychotherapie machen, liegt der Fokus klar auf „zwischenmenschlichen Faktoren“ wie z. B. der „passenden Chemie“ zwischen Therapiesuchenden und Therapeut:in und bestimmten „therapeutischen Kompetenzen“. Die Relevanz dessen spiegelt sich auch in den Ergebnissen von Felber (Felber et al. 2005), nach denen „Sympathie, Empathie und Kompetenz“ im Erstgespräch entscheidend für den Beginn einer Therapie waren. Sowohl die Anzahl an Therapiesuchenden, die sich diesbezüglich äußerten, als auch die Häufigkeit, wie oft sie dies im Interview betonten, vermitteln dabei die Relevanz dieser individuellen Affinitäten. Hinsichtlich Alters- oder Geschlechtspräferenzen äußerten mehr Therapiesuchende als beispielsweise bei Albani et al. (2010), dass sie Therapeutinnen gegenüber Therapeuten bevorzugen würden, was eher den Ergebnissen von Felber et al. (2005) entspricht. Auch hinsichtlich der Alterspräferenzen zeigte sich eine gleiche Verteilung der Präferenzen für ältere und für jüngere Therapeut:innen, wohingegen die Therapiesuchenden in der Studie von Albani und Kollegen über keine wesentlichen Alterspräferenzen berichteten (Albani et al. 2010).

Nach 3 Monaten Suche achten hingegen weniger Therapiesuchende darauf, dass „die Chemie stimmt“. Auch bezüglich des Geschlechts oder Alters des Therapeuten/der Therapeutin betrachteten Therapiesuchende ihre ursprünglichen Präferenzen nicht länger als priorisiert entscheidungsrelevant für den Suchprozess. Dies ist als kritisch zu beurteilen, da v. a. eher instinktive, teils vielleicht auch noch vorbewusste Präferenzen, z. B. die therapeutischen Passung betreffend, vernachlässigt werden. Ob dies wiederrum Auswirkungen auf das Therapie-Commitment oder die therapeutische Allianz haben könnte, ist fraglich. Dennoch wäre zu überlegen, ob und wie diesen Komponenten in zukünftigen Handlungsempfehlungen bezüglich der Reform bzw. in der psychotherapeutischen Praxis nachgekommen werden kann. Eine Idee der Therapiesuchenden hierzu war beispielsweise die Entwicklung einer einheitlichen digitalen Plattform, auf welcher sich Therapeut:innen mit Fotos, Kurzbeschreibungen und Schwerpunkten registrieren sollten.

Als entscheidungsrelevanter Faktor wird nach der Suche, ähnlich wie bei der Studie von Williams et al. (2016), die „Alltagskompatibilität“, z. B. im Vergleich zu „zwischenmenschlichen Komponenten“, priorisiert. Dies ist allerdings ggf. darüber erklärbar, dass es vielleicht für einige Therapiesuchende selbstverständlich erscheint, eine gewisse Alltagskomptabilität vorauszusetzen und diese daher anfänglich nicht als relevantes Suchkriterium zu nennen. Die generelle Alltagskompatibilität trägt allerdings entscheidend dazu bei, ob eine Psychotherapie überhaupt in Anspruch genommen werden kann. Die Gewährleistung einer flächendeckenden therapeutischen Versorgung, auch in ländlichen Gebieten, oder z. B. die Erwägung des weiteren Ausbaus videobasierter Psychotherapie, könnte in der Zukunft von noch größerer Bedeutung sein und sollte diskutiert werden, um den Zugang zu Psychotherapie für alle gleichermaßen zu ermöglichen.

Auffällig ist des Weiteren, dass einige Therapiesuchende berichteten, sich nicht ausreichend genug informiert zu fühlen, was z. B. die unterschiedlichen Therapieverfahren oder allgemeine Abläufe angehe. Dementsprechend falle es ihnen schwer, die Suche zu strukturieren oder Präferenzen zu generieren. Eine Möglichkeit, dieser Problematik zu begegnen, wäre ggf. die Organisation der mit der Reform neu eingeführten psychotherapeutischen Sprechstunden zu prüfen: Diese sind u. a. dafür gedacht, Informationen über die unterschiedlichen Therapieverfahren zu vermitteln, Indikationen zu prüfen und Behandlungsempfehlungen auszusprechen (Bundesministerium für Gesundheit 2017). Zeitlich gesehen müssen Therapiesuchende aber bereits den Suchprozess in die Wege geleitet haben, bevor Sprechstunden stattfinden können. Überlegenswert wäre die Einrichtung einer zentralen Erstanlaufstelle, in welcher Aufklärung und Informationsvermittlung, vielleicht im Rahmen der psychotherapeutischen Sprechstunden (oder anderweitig), stattfinden können. Bestenfalls könnten diese Beratungsstellen dann sogar so erweitert werden, dass darüber auch die Vermittlung von Erstgesprächen, probatorischen Sitzungen oder Therapien organisierbar wäre.

Die Ergebnisse der Studie zeigen leider deutlich, wie wenig Therapiesuchende ihr Recht auf eine „freie Wahl von Ärzt:innen/Therapeut:innen“ umsetzen können und sogar individuelle Therapiepräferenzen im Suchverlauf vernachlässigen müssen, um die Chancen auf einen Therapieplatz zu erhöhen. Die Berücksichtigung der Präferenzen im Vorfeld der Therapie ist jedoch nachgewiesen von Bedeutung für das Therapie-Commitment und bessere Behandlungsergebnisse (Swift et al. 2021). Zukünftige Reformen der Versorgungsplanung sollten als Ziel haben, Präferenzen von Therapiesuchenden besser realisieren zu können, als dies aktuell auch nach der Psychotherapiestrukturreform der Fall ist.

### Limitationen und Stärken der Studie

Eine zu betonende Stärke der Studie ist die zeitnahe und begleitende Befragung der meisten der Therapiesuchenden parallel zu ihrer Suche. So konnte z. B. die veränderte Berücksichtigung der präferierten Suchkriterien im zeitlichen Zusammenhang dargestellt werden. Gleichzeitig können durch qualitative Forschungsmethoden individuelle Perspektiven und Ansichten der Therapiesuchenden im Detail und lebensnah erfasst werden, um so Zugangsprozesse und die therapeutische Versorgungssituation aus verschiedenen Blickwinkeln abzubilden.

Die Darstellung der Ergebnisse erfolgte in Anlehnung an die COREQ-Checkliste (Tong et al. 2007). Wobei grundsätzliche Limitationen u. a. darin liegen, dass Aussagen der Therapiesuchenden inhaltlich nicht „überprüfbar“ sind. Es bleibt also z. B. unklar, wie „intensiv“ Therapiesuchende tatsächlich nach ambulanter Psychotherapie gesucht haben. Ebenso können sich ggf. die „unzureichenden Grundkenntnisse“ oder das subjektive Erleben der Therapiesuchenden in deren Aussagen spiegeln und zu verzerrten Eindrücken führen. Der daraus resultierende Konflikt entspricht jedoch zugleich den zugrunde liegenden Charakteristika und Limitationen, die mit der qualitativen Forschung einhergehen. Kritisch anzumerken ist weiterhin z. B. das unterschiedliche Maß an Vorerfahrungen sowohl mit stationärer als auch mit ambulanter Psychotherapie, welches sich z. B. auf die Präferenz des Therapeut:innen-Geschlechts auswirken kann (Speight und Vera 2005). Dies könnte sich auch in den unterschiedlichen Gruppen von Therapiesuchenden zeigen (z. B. im Hinblick auf Therapiesuchende in der Psychiatrie, die während des ersten Interviews noch stationär behandelt wurden) und sollte weiter evaluiert werden. Zu berücksichtigen ist außerdem die partielle regionale Erhebung der Stichprobe (Mainz und Umland): Würde man ländlichere Gebiete oder größere Städte betrachten, könnten sich daraus Unterschiede bzgl. der allgemeinen Versorgungssituation und dementsprechend auch bzgl. Nachfrage, Wartezeiten und womöglich der Erwartungshaltung der Therapiesuchenden ergeben (Bundespsychotherapeutenkammer 2018). Ebenso eingeschränkt ist die Repräsentativität aufgrund der eher kleinen Stichprobengröße.

Obgleich die Kernaussagen der Therapiesuchenden, aufgrund der o. g. Limitationen, gewissen Einschränkungen unterliegen, so spiegeln sie einen Konsens wider: Dieser Konsens liefert dabei Hinweise auf die, auch nach der Reform, bestehenden Schwächen hinsichtlich der Zugangs- und Versorgungssituation und die Einschränkungen, die Therapiesuchende während ihrer Suche z. B. im Hinblick auf ihre Therapiepräferenzen erleben.

## Fazit

- Obgleich belegt ist, dass sich die Berücksichtigung der Präferenzen von Therapiesuchenden positiv auf z. B. Therapieprozess und therapeutische Allianz auswirken, zeigen die Ergebnisse der Studie, dass mehr als die Hälfte der Therapiesuchenden die Suchrelevanz ihrer Präferenzen einschränkt, um so die Chance auf einen Therapieplatz zu erhöhen.
- Abgesehen davon, dass der Zugang zu Psychotherapie für alle Therapiesuchenden mit leitliniengemäßer Indikation ermöglicht und beschleunigt werden muss, sollten Lösungen gefunden werden, die Wünsche und Präferenzen der Therapiesuchenden besser zu berücksichtigen, um so das Therapie-Commitment zu stärken und den Behandlungsprozess positiv zu beeinflussen.

## References

[CR12] Albani C, Blaser G, Geyer M, Schmutzer G, Brähler E (2010). Ambulante Psychotherapie in Deutschland aus Sicht der Patienten: Teil 1: Versorgungssituation. Psychotherapeut.

[CR15] Bundesministerium für Gesundheit. Bekanntmachung eines Beschlusses des Gemeinsamen Bundesausschusses über eine Änderung der Psychotherapie-Richtlinie: Strukturreform der ambulanten Psychotherapie: BAnz AT; 2017.

[CR18] Bundespsychotherapeutenkammer (2018). Ein Jahr nach der Reform der Psychotherapie-Richtlinie: Wartezeiten 2018.

[CR10] Deutsche Gesellschaft für Psychiatrie und Psychotherapie, Psychosomatik und Nervenheilkunde, Bundesärztekammer, Kassenärztliche Bundesvereinigung, Arbeitsgemeinschaft der Wissenschaftlichen Medizinischen Fachgesellschaften, Ärztliches Zentrum Für Qualität In Der Medizin. S3-Leitlinie/Nationale VersorgungsLeitlinie Unipolare Depression – Langfassung, 2. Auflage: Deutsche Gesellschaft für Psychiatrie, Psychotherapie und Nervenheilkunde (DGPPN); Bundesärztekammer (BÄK); Kassenärztliche Bundesvereinigung (KBV); Arbeitsgemeinschaft der Wissenschaftlichen Medizinischen Fachgesellschaften (AWMF); 2015.

[CR1] Deutsche Psychotherapeuten Vereinigung (2021). 40 Prozent mehr Patientenanfragen: Corona kommt in Praxen an: DPtV-Umfrage: Hälfte wartet länger als einen Monat auf ein Erstgespräch.

[CR14] Dittrich A, Baumann E, Schlütz D, Niederberger M, Finne E (2021). Inhaltsanalyse als Methode in Prävention und Gesundheitsförderung. Forschungsmethoden in der Gesundheitsförderung und Prävention.

[CR11] Felber M, Hagleitner J, Lang M, Margreiter U, Schwentner G, Wohlgenannt M (2005). Wege zur Psychotherapie: eine Untersuchung an Psychotherapie-Klientinnen und -Klienten. Psychother Forum.

[CR6] Gelhorn HL, Sexton CC, Classi PM (2011). Patient preferences for treatment of major depressive disorder and the impact on health outcomes: a systematic review. Prim Care Companion CNS Disord.

[CR4] Glass CR, Arnkoff DB, Shapiro SJ (2001). Expectations and preferences. Psychother Theory Res Pract Train.

[CR8] Lindhiem O, Bennett CB, Trentacosta CJ, McLear C (2014). Client preferences affect treatment satisfaction, completion, and clinical outcome: a meta-analysis. Clin Psychol Rev.

[CR13] Mayring P, Fenzl T, Baur N, Blasius J (2019). Qualitative Inhaltsanalyse. Handbuch Methoden der empirischen Sozialforschung.

[CR2] Singer S, Maier L, Paserat A, Lang K, Wirp B, Kobes J (2021). Wartezeiten auf einen Psychotherapieplatz vor und nach der Psychotherapiestrukturreform. Psychotherapeut.

[CR17] Speight SL, Vera EM (2005). University counseling center clients’ expressed preferences for counselors. J College Stud Psychother.

[CR5] Swift JK, Callahan JL, Vollmer BM (2011). Preferences. J Clin Psychol.

[CR7] Swift JK, Mullins RH, Penix EA, Roth KL, Trusty WT (2021). The importance of listening to patient preferences when making mental health care decisions. World Psychiatry.

[CR16] Tong A, Sainsbury P, Craig J (2007). Consolidated criteria for reporting qualitative research (COREQ): a 32-item checklist for interviews and focus groups. Int J Qual Health Care.

[CR3] Wiegand HF, Saam J, Marschall U, Chmitorz A, Kriston L, Berger M (2020). Challenges in the transition from in-patient to out-patient treatment in depression. Dtsch Arztebl Int.

[CR9] Williams R, Farquharson L, Palmer L, Bassett P, Clarke J, Clark DM (2016). Patient preference in psychological treatment and associations with self-reported outcome: national cross-sectional survey in England and Wales. BMC Psychiatry.

